# Use of Cell Envelope Targeting Antibiotics and Antimicrobial Agents as a Powerful Tool to Select for Lactic Acid Bacteria Strains With Improved Texturizing Ability in Milk Fermentations

**DOI:** 10.3389/fbioe.2020.623700

**Published:** 2021-01-13

**Authors:** Kim I. Sørensen, Inge Kjærbølling, Ana Rute Neves, Ronnie Machielsen, Eric Johansen

**Affiliations:** ^1^Discovery, Research and Development, Chr. Hansen A/S, Hørsholm, Denmark; ^2^Emerging Technology, Chr. Hansen A/S, Hørsholm, Denmark

**Keywords:** antimicrobial agents, resistance, toolbox, improved texture, lactic acid bacteria, yogurt

## Abstract

Many antibiotics and antimicrobial agents have the bacterial cell envelope as their primary target, interfering with functions such as synthesis of peptidoglycan, membrane stability and permeability, and attachment of surface components. The cell envelope is the outermost barrier of the bacterial cell, conferring protection against environmental stresses, and maintaining structural integrity and stability of the growing cell, while still allowing for required metabolism. In this work, inhibitory concentrations of several different cell envelope targeting antibiotics and antimicrobial agents were used to select for derivatives of lactic acid bacteria (LAB) with improved properties for dairy applications. Interestingly, we observed that for several LAB species a fraction of the isolates had improved milk texturizing capabilities. To further improve our understanding of the mechanisms underlying the improved rheology and to validate the efficacy of this method for strain improvement, genetic and physiological characterization of several improved derivatives was performed. The results showed that the identified genetic changes are diverse and affect also other cellular functions than the targeted cell surface. In short, this study describes a new versatile and powerful toolbox based on targeting of the cell envelope to select for LAB derivatives with improved phenotypic traits for dairy applications.

## Introduction

Lactic acid bacteria (LAB) have been used for millennia for the fermentation of food and feed. In the dairy industry, they are used for the production of cheese and fermented milk products such as yogurt and buttermilk. The LAB not only contribute with taste and texture to dairy products, they also help to preserve them by lowering the pH, producing inhibitory compounds and through competitive exclusion of spoilage organisms (Siedler et al., 2020). Today, most food fermentations are initiated by the addition of commercial starter cultures and there is a constant need for new cultures with improved properties (Derkx et al., 2014; Johansen, 2018). One major trend in yogurt products is high texture and yogurt producers strive to make yogurt with the desired texture without the addition of thickening agents. Thus, a starter culture with the ability to improve texture of fermented milks would be a desirable solution and help manufacturers reduce cost while maintaining a simple product label. While modern biotechnology has developed multiple tools for strain improvement, public concerns about the use of gene technology in the food chain have resulted in a need to develop alternate methods of strain improvement (Johansen, 2017). These include the use of natural selection and evolution (Johansen, 2018) as well as the use of dominant selection, for example, based on resistance to inhibitory substances (Derkx et al., 2014; Cardoso et al., 2018).

**FIGURE 1:**
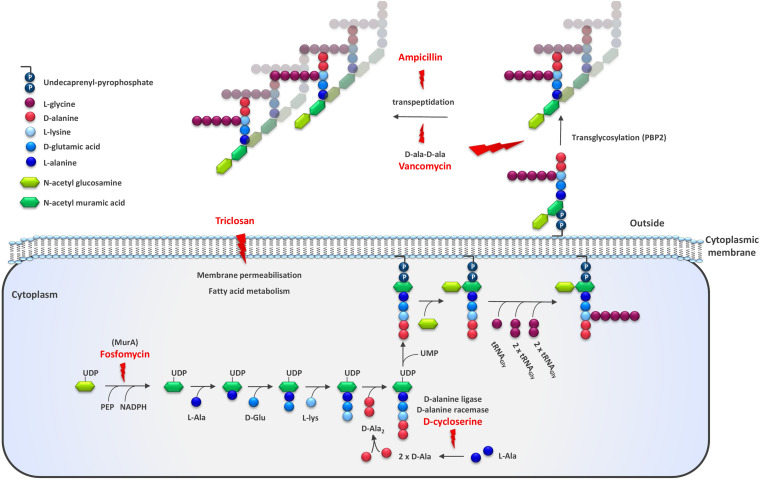
Schematic representation of the enzymatic steps involved in Gram positive cell wall synthesis. The targets of cell envelope targeting antibiotics and antimicrobial agents used for strain improvement are indicated with a lightning symbol. The illustration is adapted from McCallum et al. (2011) and depicts the cell wall of *Staphylococcus aureus*. Among the different LAB species, the nature of interpeptide cross-bridge, the fifth residue stem peptide, the glycan chain length, and other features may vary.

Antimicrobial agents have been developed primarily for the control of pathogenic organisms. They have a specific mode-of-action, targeting a specific component of bacterial metabolism. However, since these components are common to a wide range of microbes, most antimicrobials also inhibit non-pathogens. One feature of the bacterial cell that is commonly targeted directly by antimicrobials is the cell surface and especially the assembly of the peptidoglycan layer. Ampicillin (AMP) acts on peptidoglycan crosslinking, inhibiting the transpeptidase responsible for the crosslinking, while vancomycin binds the D-ala-D-ala terminal of the growing peptidoglycan chain, inhibiting both the polymerization and cross-linking steps (Barna and Williams, 1984) (Figure 1). Other antimicrobials interfere with the synthesis of peptidoglycan precursors in the cytosol. For example, D-cycloserine (DCS) targets two enzymes, alanine racemase and D-ala:D-ala ligase, involved in the production of D-ala-D-ala which forms the terminal end of the UDP-MurNAc pentapeptide while fosfomycin targets UDP-*N*-acetylglucosamine enolpyruvyl transferase, the first committed step in peptidoglycan biosynthesis (Lambert and Neuhaus, 1972; Prosser and de Carvalho, 2013). Triclosan provokes oxidative stress in bacteria and damages the cell membrane (Lu et al., 2018). We have previously characterized several LAB variants spontaneously resistant to DCS and AMP (Kibenich et al., 2012; Johansen et al., 2013). Some of these derivatives showed reduced whey syneresis as well as increased texture formation when grown in milk compared to the parent strain. The other growth properties were found to be unaffected relative to that of the parent strain. Subsequently, we wanted to explore the potential of using other cell envelope targeting antimicrobials for the selection of strains with improved texture in milk. We therefore applied a dominant selection strategy based on resistance to several different antimicrobials to generate a strain development toolbox for improved rheological properties. Here we describe the characterization of the obtained resistant derivatives with improved texture for dairy application.

## Materials and Methods

### Bacteria and Growth Conditions

All bacterial strains are listed in Table 1. Wild-type strains are from the Chr. Hansen culture collection (Johansen et al., 2015) and were originally isolated from dairy cultures or dairy products.

**TABLE 1:** Strain list and texture properties.

*Streptococcus thermophilus*		Relative shear stress*	Relative gel firmness*
CHCC13994	Wild-type	100	100
CHCC13235	Cycloserine resistant derivative of CHCC13994	93	121
CHCC13236	Cycloserine resistant derivative of CHCC13994	114	119
CHCC15914	Wild-type	100	100
STFOS92	Fosfomycin resistant derivative of CHCC15914	111	113
STVAN16	Vancomycin resistant derivative of CHCC15914	100	120
CHCC27806	Wild-type	100	100
STTRI97	Triclosan resistant derivative of CHCC27806	99	103
STVAN19	Vancomycin resistant derivative of CHCC27806	100	123
STVAN20	Vancomycin resistant derivative of CHCC27806	108	115
STVAN24	Vancomycin resistant derivative of CHCC27806	100	103
***Lactobacillus delbrueckii* subsp. *bulgaricus***			
CHCC13995	Wild-type	100	100
CHCC12944	Cycloserine resistant derivative of CHCC13995	109	150
CHCC12945	Cycloserine resistant derivative of CHCC13995	110	158
CHCC15461	Ampicillin resistant derivative of CHCC13995	101	134
CHCC15466	Ampicillin resistant derivative of CHCC13995	100	114
CHCC759	Wild-type	100	100
CHCC15470	Ampicillin resistant derivative of CHCC759	109	105

*For each group of strains, the values are relative to the respective parent strain. The absolute values for the wild-type strains are in Pascal units (shear stress, gel firmness): CHCC13994 (70, 137), CHCC15914 (83, 225), CHCC27806 (77, 229), CHCC13995 (99, 59), and CHCC759 (45, 199).

*Lactobacillus delbrueckii* subsp. *bulgaricus* strains were cultured using MRS medium (de Man et al., 1960) (Oxoid) at 37°C. Liquid cultures were propagated without shaking; plate cultures (1% w/v agar; Oxoid) were incubated in anaerobic jars (Anaerocult A, Millipore). *Streptococcus thermophilus* strains were grown in M17 medium (Terzaghi and Sandine, 1975) (Difco Laboratories) with 2% (w/v) lactose, under the same environmental conditions. Milk fermentations for screening purposes were carried out in B-milk, which consists of skim milk powder at a level of dry matter of 9.5% (w/v) reconstituted in distilled water and pasteurized at 99°C for 30 min, followed by cooling to 30°C (Sørensen et al., 2016). Full fat milk with 2% added whey protein was used for rheologic characterization of selected strains. Full fat (whole) cow’s milk contains 3.5% fat and was obtained from Arla Food Ingredients.

### Selection of Antimicrobial Resistant Isolates

To identify a suitable concentration of an antimicrobial agent for selection of resistant isolates, a single colony of the parental strain was inoculated into the preferred broth containing various concentrations of the antimicrobial and incubated 20 h. The suitable concentration was typically identified as the concentration that reduced the OD_600_ measured growth, after 20 h growth at 37°C. The suitable concentration was identified as the concentration that reduced the OD_600_ measured after 20 h to approximately 20% of that obtained during growth in the same medium without added antibiotic. A standardized approach was used for the isolation of variants. A single colony of the parent strain was inoculated into the appropriate broth containing the antimicrobial agent at the predetermined suitable concentration and incubated to saturation. Saturated cultures were plated on appropriate agar without the antimicrobial agent. Isolated colonies were picked and screened in microtiter plates (Johansen et al., 2015) for the ability to grow in the presence of the antimicrobial agent at the selective concentration. Typically, 25% of the resistant isolates were identified as fast growing derivatives. After purification, the isolates were screened for their basic texturizing properties when grown in B-milk (Kibenich et al., 2012; Johansen et al., 2013). The selective concentrations of the antimicrobial agents were: DCS, 50 or 100 μg/ml; AMP, 0.3 or 0.5 μg/ml; fosfomycin, 70 μg/ml; vancomycin, 0.05 μg/ml; and triclosan, 4 μg/ml. All antimicrobials were from Sigma–Aldrich.

### Texturizing Properties of Antibiotic Resistant Derivatives in Milk

The screening of the selected resistant derivatives was performed using two different approaches. DCS and AMP resistant derivatives, showing improved rheological properties, were identified as having reduced whey syneresis and perceived improved texture when grown in milk compared to the parent strain (Kibenich et al., 2012). While to identify the fosfomycin, vancomycin, and triclosan resistant derivatives with improved texturizing properties, the selected isolates were grown to saturation in broth and inoculated 1% vol/vol into 96-well deep-well plates containing 1.8 ml/well B-milk including the pH indicators bromocresol purple and bromocresol green (Poulsen et al., 2019). After fermentation at 37°C, the texturizing properties of the derivatives were assessed using the TADM-based method described in Poulsen et al. (2019). Finally, the most interesting candidates from all selections were grown to saturation in the appropriate broth and inoculated 1% vol/vol into 200 ml full fat milk and then fermented overnight at 40°C. The milk used for rheology measurement was added 2% whey protein to standardize the protein content of the milk. Acidification was followed using a CINAC system (Corrieu et al., 1992) or an ICINAC system (AMS alliance). Fermentation was stopped at pH 4.55 and the contents of the bottle were carefully stirred and placed at 6°C overnight before rheology measurements. Rheologic properties were measured in a StressTech rheometer (RheoLogica Instruments AB, Sweden) equipped with a C25 coaxial measuring system (Folkenberg et al., 2011). The viscometry test was made with shear rates varying from 0.27 to 300 s^–1^ in 21 steps. Shear rates were increased and then decreased and the upward and downward curves of shear stress and apparent viscosity were recorded. Delay and integration times were 5 and 10 s, respectively. Shear stress at 300 s^–1^ was chosen as a standard for comparisons. G^∗^, reflecting gel firmness, was measured by oscillation at frequency of 1 Hz. We found that among the fast growing isolates, 1–5% were found to have improved texturizing properties. The rheological data of the strains studied here are presented in Table 1. For most strains, the values represent an average of duplicate experiments but due to milk variations over the experimental period, the values for each group of strains are set relative to that of the respective parent strain.

### Genome Sequencing and SNP/INDEL Analysis

Genomic DNA for *de novo* short-read whole genome sequencing (WGS) was extracted from 1 ml of overnight culture (at OD_600_ = 1) of wild-type and mutant strains with DNeasy Blood and Tissue kit on QiaCube system (Qiagen) following the manufacturer’s protocol. Sequencing was done at BGI using Illumina Hiseq 2000 machines or in-house at Chr. Hansen using an Illumina MiSeq machine. The genome sequences were assembled, annotated, and finished using CLC genomic workbench software (CLCBio). Reference-quality hybrid genome assemblies were created for selected strains using the Oxford Nanopore Technologies (ONT) long reads and the corresponding Illumina short reads. Poor quality reads (≤30 bases, ≤quality 20) were trimmed using AdapterRemoval v.2.2.4 based on the PHREDscale. The hybrid genome assembler Unicycler v.0.4.7 was used for the *de novo* assembly of the trimmed short reads and the raw ONT reads. In order to reduce sequence misassembly, the non-default parameter: “conservative” was selected for the hybrid assembly. The annotated genome sequences of all variant strains were aligned and compared with the respective mother strains using Mauve 2.3.1 software. After alignment, a single-nucleotide polymorphism (SNP) analysis was performed using Mauve 2.3.1 software. Breseq v. 0.35.0 was used as a secondary method to assess SNPs, small deletions, and insertions by mapping reads from variant sequences to the reference genome of the mother strain (Deatherage and Barrick, 2014). Subsequently, gdtool was used to compare and collect the identified SNPs. The similarities with identified genes from other organisms available in GenBank^1^ were assessed by using the Basic Local AlignmentSearchTool (BLAST) (Altschul et al., 1990), provided by the National Center for Biotechnology Information^2^.

### Intracellular Magnesium and Manganese Concentrations

**FIGURE 2:**
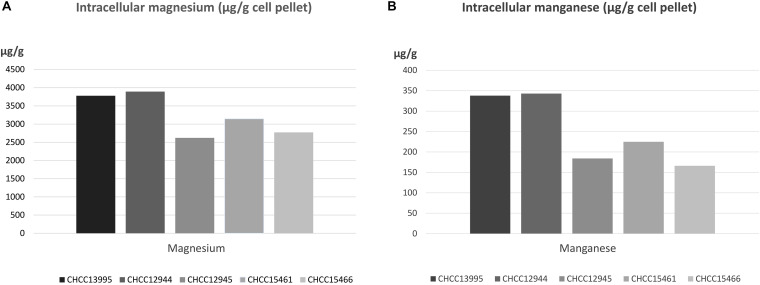
Intracellular levels of **(A)** magnesium (μg/ml) and **(B)** manganese (μg/ml) measured in wild-type strain CHCC13995 and texturizing derivatives CHCC12944, CHCC12945, CHCC15461, and CHCC15466 measured by inductively coupled plasma–mass spectrometry (ICP-MS) analysis.

To prepare the cellular extracts, fresh overnight cultures in MRS were inoculated at 1% (vol/vol) into 100 ml MRS and incubated anaerobically overnight at 37°C. Cultures were then split into two cultures of 50 ml and cells were collected by centrifugation at 5000 r/min for 10 min (A and B samples for each strain). After first centrifugation, the cells were washed once at 4°C with MRS and then twice with 5 ml 1x phosphate buffered saline (PBS) buffer (pH 7.4). The PBS buffer had been treated overnight with 10 mg/ml Chelex (Bio-Rad Inc.). All tubes were then centrifuged for 10 min at 5000 r/min, and washed with 5 ml PBS buffer, centrifuged for 10 min at 5000. After washing and resuspension in 1 ml PBS buffer, the contents were transferred to 2 ml Eppendorf tube. The Eppendorf tubes were centrifuged for 15 min at 15,000 x *g* and the supernatants were discarded. The cell pellets were dried in a rotary evaporator at room temperature, weighed and resuspended in 200 μl (vol/vol) concentrated (65%) nitric acid containing 0.1% Triton-X 100. The tubes were incubated at 95°C with shaking for 10 min followed by vigorous vortexing for 20 s. Finally, the tubes were centrifuged at 15,000 x *g* for 5 min and the supernatants transferred to new tubes. The supernatants for each strain (samples A and B) were sent to Hans van der Velde, Micro-analyse, Rijksuniversiteit Groningen, Netherlands where they were analyzed for total content of magnesium and manganese using inductively coupled plasma mass spectroscopy (ICP-MS) using a Perkin Elmer Optima 7000 DV (Wilschefski and Baxter, 2019). Both extractions showed the same tendency but only sample B was sufficiently extracted and these data are presented in Figure 2.

### EDTA Chelating Experiments

The CHCC13995 wild-type strain and derivatives, CHCC12945, CHCC12944, CHCC15461, and CHCC15466 were grown anaerobically at 40°C for approximately 20 h and inoculated 1% vol/vol into 200 ml B-milk. The cultures were incubated overnight at 40°C and the development of pH was followed continuously over approximately 20 h using an ICINAC system (AMS alliance). When used, EDTA (Sigma–Aldrich) was added to a final concentration of 100 μg/ml, MgSO_4_ (Sigma–Aldrich) was added to a final concentration of 50 μg/ml, and MnSO_4_ was added to the final concentrations of 10, 25, 50, or 100 μg/ml.

## Results and Discussion

### Application of Antimicrobial Agents for Industrial Strain Improvement

We used a number of antimicrobials, including the antibiotics DCS, AMP, vancomycin, and fosfomycin, as well as the biocide triclosan to select resistant variants which were subsequently screened for desirable traits for industrial application. For each compound, we developed a selection protocol optimized for the particular strain and antimicrobial (for details, see section “Materials and Methods”) and obtained a suitable number of variants for screening purposes. In the dairy industry, strains with optimal rheological traits are in high demand. With this in mind, we screened all isolates obtained upon selection with antimicrobial compounds for their texturing potential in milk. The best milk texturizing strains had up to 14% improved shear stress or up to 58% higher gel firmness (Table 1). Especially gel firmness was higher in many of the selected derivatives. These strains were selected for further characterization, including WGS to identify mutations that may confer resistance to the compound of choice as well as mutations presumably involved in the improved texturizing phenotype. Data from wild-type and variant strains were compared and analyzed for non-synonymous SNPs and gaps. The outcome of this analysis is presented in Table 2.

**TABLE 2:** Gene modifications identified in resistant and texturizing derivatives.

Resistance	Species^1^	Texturizing derivative	Background	Gene modification	Gene accession^2^
Cycloserine	St	CHCC13235	CHCC13994	EAM type sugar transporter glcU (302T to 302C, V101A)	MW084972, MW084973
				LacI type regulator (−202A to −202C)	MW292712, MW292713
Cycloserine	St	CHCC13236	CHCC13994	MFS transporter (337A to 337G, I113V)	MW292666, MW292667
Cycloserine	Lb	CHCC12945	CHCC13995	Mg^2+^ transporting P-type ATPase (898T to 898A, 899T to 899A, Δ916C) Truncated	MW292668, MW292669
				PTS sugar transporter subunit IIC—cellebiose: (536C to 536G, A179G)	MW292672, MW262673
Ampicillin	Lb	CHCC15461	CHCC13995	Mg^2+^ transporting P-type ATPase (Δ181 - 184) Truncated	MW262668, MW292670
				Aquaporin/glycerol uptake facilitator. (115A to 115C, I39L)	MW292674, MW292675
Ampicillin	Lb	CHCC15466	CHCC13995	Mg^2+^ transporting P-type ATPase (2273C to 2273A, P758Q)	MW292668, MW292671
				Spermidine/putrescine ABC transporter, ATP-binding protein (254A to 254C, Y85S)	MW292677, MW292678
				Aquasporin/glycerol uptake facilitator protein (115A to 115C, I39L)	MW292674, MW292676
Ampicillin	Lb	CHCC15470	CHCC759	Beta-subunit DNA polymerase (1504G to 1504T, G502C)	MW292679, MW292680
				Tyrosine-protein phosphatase (73G to 73C, G25R)	MW292681, MW292682
Fosfomycin	ST	STFOS92	CHCC15914	UDP-*N*-acetylglucos-amine_1-carboxyvinyl-transferase – murA. (122G to 112A, E38stop). Truncated	MW292682, MW292684
				Pyruvate kinase (653G to 653A, A218T)	MW292685, MW292686
				Calcium-translocating P-type ATPase, PMCA-type (1990G to 1990A, G664R)	MW292687, MW292688
Vancomycin	ST	STVAN16	CHCC15914	PBP1A family penicillin-binding protein (966A to 966C, E322D)	MW292689, MW292690
				LysM peptidoglycan-binding domain-containing protein (541T to 541C, S181P)	MW292691, MW292692
				Anthranilate phosphoribosyltransferase. (979A to 979C, R327S)	MW292693, MW292694
				Hypothetical protein (1630A to 1630T, S544C)	MW292695, MW292696
				Glycine-tRNA ligase subunit alpha (438C to 438A, F146L)	MW292697, MW292698
Vancomycin	St	STVAN19	CHCC27806	Large subunit carbamoyl-phosphate synthase (452C to 452T, T151M)	MW292699, MW292700
Vancomycin	St	STVAN20	CHCC27806	Dihydroxyacetone kinase (140C to 140T, T47I)	MW292701, MW292702
				Cell wall CHAP hydrolase (−67C to −67T)	MW292703, MW292704
				Response regulator transcription factor (lytR family). T insertion at −109	MW292705, MW292706
Vancomycin	St	STVAN24	CHCC27806	Glycosyltransferase polysaccharide biosynthesis gene—rgp. (282G to 282A, M94I)	MW292707, MW292708
				Ribosome silencing factor. (166C to 166T, R56C)	MW292709, MW292710
Triclosan	St	STTRI97	CHCC27806	Glutathione-disulfide reductase (104G to 104A, G35E)	MW292711, MW292712

*^1^St (*S. thermophilus*); Lb (*L. bulgaricus*). ^2^Accession numbers: Background strain, derivative. Nucleotide positions: +1 defines A in AUG start codon.*

### Genetic Changes in Derivatives Obtained After Antimicrobial Selection

#### Analysis of Genetic Changes in *Streptococcus thermophilus* Strains Resistant to D-Cycloserine

Two *S. thermophilus* strains with improved texture formation were isolated among DCS resistant derivatives (Kibenich et al., 2012; Johansen et al., 2013). Genome sequence analysis of these derivatives, strains CHCC13235 and CHCC13236, revealed three non-synonymous SNPs resulting in amino acid replacement in the encoded proteins, possibly altering the enzyme activity and therefore potentially involved in the observed DCS resistance. CHCC13235 has an SNP in a gene annotated as EAM type sugar transporter (glcU) (302C to 302T, resulting in amino acid change V101A). GlcU encodes a novel non-PTS glucose permease (Castro et al., 2009), which previously has been shown to play a role in fitness cost in nisin-resistant variants of *Streptococcus faecalis* (Kumar et al., 2019). In addition, CHCC13235 carries an SNP in the promoter region upstream of a LacI type regulator potentially regulating the neighboring gene maltodextrin phosphorylase, whose role in milk fermentations is unknown but was previously found to be upregulated in *S. thermophilus* under acidic growth conditions (Wu et al., 2019). CHCC13236 has an SNP in a gene encoding an MFS transporter (nucleotide change A337 to G337, resulting in amino acid change I113V). Thus, both strains have non-synonymous substitutions in a protein with a transporter function which have previously been suggested to be involved in resistance to DCS via an antibiotic efflux mechanism (Versluis et al., 2016). No direct linkage to the observed improved texturizing properties could be identified.

#### Analysis of Genetic Changes in *L. delbrueckii* subsp. *bulgaricus* Strains Resistant to D-Cycloserine and Ampicillin

The most texturizing derivative of CHCC13995 is the DCS resistant CHCC12945. This strain has T to A substitutions at positions 897 and 898, and a 1 bp deletion at nucleotide position 899 of a gene encoding a Mg^2+^ transporting P-type ATPase, while CHCC15461, an AMP resistant derivative, was found to have a 4 bp deletion in the same gene starting at nucleotide position 181. In both cases, the change results in premature termination of the protein. In addition, CHCC12945 harbors an SNP in a gene encoding a PTS sugar (cellobiose) transporter subunit IIC (nucleotide change 536C to 536G, resulting in the predicted amino acid sequence alteration A179G). Chen et al. (2017) also found a sugar transporter encoding gene among the novel genes conferring DCS resistance. Potentially, the sugar transporter can mediate transport of DCS facilitating contact with the target enzymes (Versluis et al., 2016; Chen et al., 2017). Strain CHCC15466, an AMP resistant derivative, has an SNP in the same ATPase (nucleotide change 2273C to 2273A, resulting in P758Q). In addition, the two AMP resistant isolates, CHCC15461 and CHCC15466, both have the identical SNP in a gene annotated as encoding a glycerol uptake facilitator protein GlpF (nucleotide change 115A to 115C, resulting in I39L) while in CHCC15466, another SNP was identified in a polyamine (spermidine/putrescine) ABC transporter encoding gene (254Ato 254C, Y85S). In *Staphylococcus aureus*, the GlpF level correlates with formation of the L-form of the bacteria which is more tolerant to antibiotics such as AMP and secretes more exopolysaccharide (EPS) (Han et al., 2014). If a similar mode of action exists in *L. delbrueckii* subsp. *bulgaricus*, the amino acid change predicted should result in a more active GlpF protein. Interestingly, exposure of *Streptococcus pneumoniae* to penicillin resulted in down-regulation of the polyamine transporter (Rogers et al., 2007) while spermidine was found to stimulate biofilm formation in *Bacillus subtilis* (Hobley et al., 2017).

In another *L. delbrueckii* subsp. *bulgaricus* strain background, CHCC759, a more texturizing and AMP resistant derivative was also selected, CHCC15470. This derivative had no SNP in the cation transporting P-type ATPase but instead we identified an SNP in a gene encoding a tyrosine-protein phosphatase (73G to 73C, resulting in aa change G25R) and in the beta-subunit of DNA polymerase (1504G to 1504T, resulting in aa change G502C) (Table 2). While the relationship between changes in the DNA polymerase and improved texture or antibiotic resistance is not apparent, tyrosine-protein phosphatases have been shown to be involved in the regulation of extracellular polysaccharide synthesis and assembly (Vincent et al., 2000). Strains with higher extracellular polysaccharide production have been found to exhibit a significantly higher resistance to antibiotics (Arciola et al., 2005) and extracellular polysaccharide is known to be involved in texture formation in dairy products.

Interestingly, three derivatives of *L. delbrueckii* subsp. *bulgaricus* strain CHCC13995 with improved texturizing properties were found to have genetic changes, at different positions, in the same gene; a gene annotated as a Mg^2+^ transporting P-type ATPase (*mgtA*). Two of these derivatives, CHCC15461 and CHCC15466, were selected as resistant to AMP and one strain, CHCC12945, as DCS resistant. A more thorough characterization of these derivatives was therefore carried out as described in a subsequent section.

#### Analysis of Genetic Changes in Strains Resistant to Vancomycin

Vancomycin was used to select a number of derivatives with improved rheological properties in two different background strains of *S. thermophilus*, CHCC15914 and CHCC27806. Table 2 presents the changes detected in four vancomycin resistant derivatives of *S. thermophilus* showing improved rheological properties. Three of the derivatives, STVAN19, STVAN20, and STVAN24 are derived from CHCC27806. STVAN19 contains a single SNP present in the *carB* gene encoding the large subunit of carbamoyl phosphate synthetase (452C to 452T, resulting in aa change T151M), which is located in ATP-grasp fold 1 of this subunit. It is not known if this change results in a more or less active enzyme. Interestingly, vancomycin binds to D-alanyl-D-alanine and the active site of CarAB is very similar to that previously described for D-alanine:D-alanine ligase (Stapleton et al., 1996). In addition, *carB* was found to be the most highly induced gene in a vancomycin sensitive *S. aureus* strain when grown in the presence of vancomycin (Awad et al., 2013) and has previously been found to be involved in multiple cellular functions including biofilm formation and EPS formation (Zhuo et al., 2015). In STVAN20, we identified four SNPs with two occurring in promoter regions of different genes. These are: a cell wall CHAP hydrolase (C to T change at position −67 from ATG start codon) and a transcription factor annotated as a response regulator (a T insertion in a long row of T’s at −109 from ATG start codon). In both cases, such promoter changes may alter the level of expression for the downstream genes. The CHAP hydrolases play a role in cell wall biosynthesis which could affect vancomycin resistance and trigger EPS production (Monteiro et al., 2017). Response regulators have been shown to regulate vancomycin resistance in *E. faecium* (Haldimann et al., 1997). The third SNP was found in the coding region of the *dak* gene encoding the cytoplasmic enzyme dihydroxyacetone kinase (140C to 140T, T47I). This change might result in an altered activity of the enzyme. A potential *dak* gene has been identified as a target for vancomycin in *S. aureus* (Haag et al., 2015) and DHA-induced genes have been found to be involved in a biofilm growth state in *E. coli* (Peiro et al., 2019). The last vancomycin resistant derivative in the CHCC27806 background, STVAN24, was found to contain two SNPs. One SNP was identified in a glycosyltransferase polysaccharide biosynthesis gene *cspB* (282G to 282A, M94I) and the second occurred in a gene predicted to encode a ribosome silencing factor (166C to 166T, R56C). Both are amino acid changes that are likely to change the activity of their respective encoded proteins. Only the change in *cspB* is expected to be directly involved in polysaccharide biosynthesis and texture formation (Schmid et al., 2016).

Finally, strain STVAN16 derived from the CHCC15914 strain background, was found to possess 5 different SNPs, all within the coding region of the encoded protein. SNP1 is in the gene encoding PBP1A family penicillin-binding protein (966A to 966C, E322D), SNP2 in the gene encoding LysM (543T to 543C, S191P), SNP3 is in a gene encoding Anthranilate phosphoribosyltransferase (979A to 979C, R327S), SNP4 occurred in a hypothetical protein (1630A to 1630T, S544C) while the last SNP in the alpha subunit of Glycine-tRNA ligase (438C to 438A, F146L).

Only the first two of these five genes have previously been associated with vancomycin resistance and texturizing properties; the PBP1A family penicillin-binding protein and LysM peptidoglycan-binding domain-containing protein. Both proteins are involved in cell wall biosynthesis and are induced in the vancomycin resistant *Enterococcus faecium* strain AUS0004 (Cacaci et al., 2018). Furthermore, disruption of the gene encoding penicillin-binding protein 2b was resulted in reduction of EPS production in *S. thermophilu*s Sfi6 (Stingele and Mollet, 1996). No direct correlation to vancomycin resistance or texture could be made for the three other SNPs in STVAN16.

#### Analysis of Genetic Changes in a Strain Resistant to Fosfomycin

From CHCC15914, one fosfomycin resistant derivative with improved texturizing properties was isolated. The strain, STFOS92, contains three SNPs (Table 2). SNP1 results in a truncated UDP-*N*-acetylglucosamine-1-carboxyvinyltransferase protein due to a nucleotide change resulting in premature translation termination after amino acid 38E, SNP2 is in the gene encoding pyruvate kinase (653G to 653A, A218T) while SNP3 occurs in a gene annotated as a calcium-translocating P-type ATPase, PMCA-type (1990G to 1990A, G664R). From these three observed changes, only the change in *murA* is expected to be associated with the observed phenotype as MurA catalyzes the first step in the biosynthesis of the bacterial cell wall and is a target of fosfomycin (Figure 1). Furthermore, it is likely that the potential accumulation of the EPS precursor UDP-N-acetyl-alpha-D-glucosamine leads to increased formation of EPS (Zeidan et al., 2017). This derivative was found to aggregate significantly.

#### Analysis of Genetic Changes in a Strain Resistant to Triclosan

One triclosan resistant derivative with improved texturizing properties was isolated. The strain, STTRI97, a derivative of CHCC27806, contains an SNP in the gene encoding glutathione-disulfide reductase (nucleotide change G104A resulting in amino acid change G35E) (Table 2). Triclosan provokes oxidative stress and damages the bacterial membrane (Lu et al., 2018). A change in glutathione disulfide reductase activity may help the bacteria to overcome this oxidative damage (Patel et al., 1998).

### Intracellular Magnesium and Manganese Concentrations of Variants With Changes in the P-Type ATPase

The observation that three of the DCS or AMP resistant isolates, CHCC12945, CHCC15461, and CHCC15466, carried mutations in the same gene, annotated as encoding a Mg^2+^ transporting P-type ATPase, suggested an inactivation of function which could lead to a lower intracellular magnesium concentration. Furthermore, we speculated that a lower intracellular concentration of magnesium could play a role in texture formation and/or resistance to the two antimicrobial agents. Cell pellets from the individual cultures were therefore subjected to ICP-MS analysis of the total intracellular magnesium and manganese content. We included another DCS resistant mutant, CHCC12944, that shows improved texture but has no genetic changes in the ATPase. Surprisingly, the data obtained from the analysis showed that the ATPase variants all have reduced intracellular levels of both magnesium and manganese when compared to the parental strain (CHCC13995) and the non-ATPase derivative CHCC12944. The reduction in intracellular levels of magnesium ranged from 17% in CHCC15461 and up to 31% in CHCC12945 and the reduction in manganese levels was from 33% (CHCC15461) and up to 51% (CHCC15466) when compared to the levels found for the CHCC13995 mother strain. The intracellular level of magnesium is approximately 10-fold higher than for manganese.

#### EDTA Inhibits and Mn^2+^ Restores Milk Acidifications of the P-Type ATPase Variants

**FIGURE 3:**
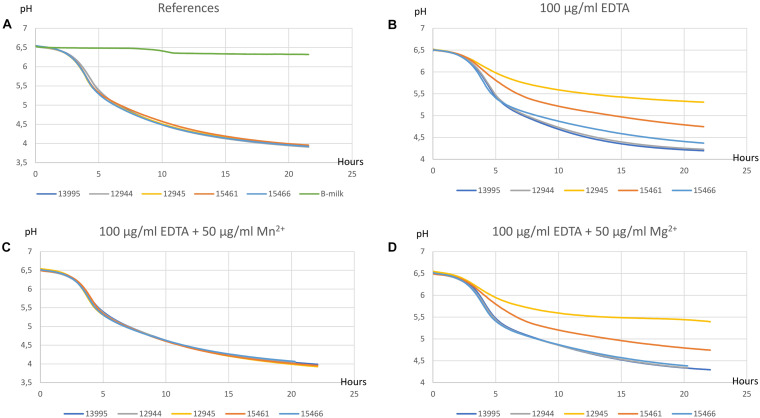
Milk acidification curves. All experiments were performed for wild-type strain CHCC13995 and texturizing derivatives CHCC12944, CHCC12945, CHCC15461, and CHCC15466 at 40°C in B-milk **(A)**, with 100 μg/ml EDTA **(B)**, with 100 μg/ml EDTA + 50 μg/ml Mn^2+^ **(C)**, and with 100 μg/ml EDTA + 50 μg/ml Mg^2+^ added **(D)**.

EDTA is a chelating agent with high affinity for calcium, magnesium, and other divalent metal cations. The effect of addition of EDTA to milk acidifications was assessed for the three ATPase variants, the DCS resistant mutant CHCC12944, and the CHCC13995 mother strain. The effect of adding various concentrations of Mg^2+^ and Mn^2+^ to milk containing 100 μg/ml EDTA was also determined. The pH achieved after 20 h incubation is shown in Figure 3. In the absence of EDTA, a pH of 4.0 was attained after 20 h for all strains (Figure 3A). Addition of 100 μg/ml EDTA inhibited the ATPase variants more than CHCC12944 and CHCC13995 (Figure 3B). Addition of 50–200 μg/ml MgSO_4_ (only 50 μg/ml is shown) did not restore the EDTA inhibited acidification but had a minor effect on acidification of CHCC15466 at 50 μg/ml (Figure 3D). On the other hand, addition of 10 μg/ml MnSO_4_ partly restored the acidification of the mother strain and for derivatives CHCC15461 and CHCC15466 but to a lesser extent for the derivative CHCC12945 (not shown). Addition of 50 μg/ml MnSO_4_ completely restored acidification of both mother strain and the derivatives (Figure 3C). Addition of 50 μg/ml MgSO_4_ to cultures supplemented with MnSO_4_ had no additional effect (not shown).

#### P-Type ATPase—A Role for Antibiotic Resistance and Texture?

P*-*type ATPases transport a variety of different compounds, including ions and phospholipids, across a membrane using ATP hydrolysis for energy (Axelsen and Palmgren, 1998; Chan et al., 2010). The MgtA and MgtB ATPase type, described here, are normally associated with active influx of Mg^2+^, which is the most abundant divalent intracellular cation and a cofactor with ATP in many enzymatic reactions (Smith and Maguire, 1998). Manganese transporting P-type ATPases are not well known but at least one was described for mycobacteria (Padilla-Benavides et al., 2013*).* Instead, two other manganese uptake systems have been reported for LAB: the NRAMP-type transporter MntH and the ABC transporter SitABC (Kehres and Maguire, 2003). MntH is the major transporter under acidic conditions while SitABC is active at neutral pH (Porcheron et al., 2013; Siedler et al., 2020). Mn^2+^, an important trace element, also plays a role in bacterial physiology and protection against oxidative stress (Kehres and Maguire, 2003).

The genetic changes detected in the variants described here are expected to either inactivate the ATPase completely due to premature translation termination (CHCC12945 and CHCC15461) or reduce the activity (CHCC15466). All derivatives were found to have lower intracellular levels of both magnesium and manganese when grown in standard medium. Another DCS resistant and texturizing derivative with no genetic change in the ATPase encoding gene, CHCC12944, had levels of magnesium and manganese similar to the mother strain CHCC13995. The addition of EDTA to milk inhibited acidification of the ATPase variants to a greater extent than the other derivatives and the mother strain. The EDTA mediated inhibition was restored by addition of Mn^2+^ while addition of Mg^2+^ had only minor effect. We interpret these results to indicate that the change in the ATPase leads to an intracellular Mn^2+^ deficiency in the presence of EDTA while the intracellular reduction in Mg^2+^ is less critical. Together, these results suggest that the P-type ATPase is a not a specific Mg^2+^ transporter but rather a transporter of both Mg^2+^ and Mn^2+^ and potentially other cations. Interestingly, the most texturizing derivative, CHCC12945, is more inhibited by EDTA than the other derivative with a truncated ATPase, CHCC15461. This could indicate that the potential PTS sugar transporter, found to be altered in CHCC12945, also plays a role in texture formation. Whether the reduced intracellular levels of magnesium and manganese are directly or indirectly related to the observed antibiotic resistance and observed texture improvement is unclear but the only genetic change common to these three derivatives is in the gene encoding the P-type ATPase. The results from Lew et al. (2013) indicated that the ratio between manganese and magnesium ions in *Lactocaseibacillus rhamnosus* affected biosynthesis of lipoteichoic acid and these authors discussed whether the ratio between the metals indirectly affected the level of peptidoglycan by modifying the activity of the enzymes MurA to MurF. Similarly, the target for DCS, D-ala:D-alanine ligase, can itself be affected. Finally, it can be speculated that the P-type ATPase plays a more direct role in the uptake of DCS and AMP but this seems unlikely due to the different nature of the two antibiotics.

## Conclusion

We have presented a new powerful toolbox to select for strains with improved texture formation in milk. By using inhibitory concentrations of different cell envelope targeting antibiotics and antimicrobial agents to select variants, we were able to identify a number of isolates that showed improved rheological properties in milk. Surprisingly, the characterization of these derivatives showed that the identified genetic changes occurred, in only a few cases, in target genes typically associated with resistance to the antimicrobial compounds. The versatility of this method and the diversity of mutations detected were unexpected. Therefore, we also expect this tool to be useful to select for other improved traits in bacteria used for dairy and other fermentation applications. Together these results show that mechanisms leading to resistance to antibiotics and antimicrobials are complex and we speculate this to be dependent on the growth medium used during the application of these dominant selection strategies. Similar observations have also been made by others (Baisa et al., 2013.) who described that individual resistance mechanisms are typically well defined in minimal and defined medium but more complex in rich media and can involve several different genetic loci. This highlights the necessity of understanding the mode of action for each specific antibiotic and antimicrobial agent in a particular medium, especially when new antibiotic targets are to be identified for the prevention and treatment of disease. In our case, the selections were done in complex media like M17 and MRS medium and the genetic characterization of the selected resistant derivatives led to the discovery of mutations in a variety of different genes. The characterized resistant strains generally comprise mutations in genes associated with transport functions, formation of the outer surface of the cell or stress responses. Some of the genes have not previously been associated with resistance to the compounds used. However, our results will only partly reflect the resistance mechanism to be found in the complex media used in these studies because the isolates investigated were a subset of the total number of resistant derivatives, specifically selected for their improved texturizing ability. This also complicates the understanding of how the genetic changes link to the observed resistance and texturizing phenotype because the two phenotypes are not separated. This is especially the case when multiple genetic changes occur in an isolate. In a number of cases, there was a predictable link to the improved texturizing phenotype that was observed. For example, in the AMP^R^ derivative CHCC15470 and the VAN^R^ derivative STVAN24, SNPs were identified in genes encoding a tyrosine-protein phosphatase and a glycosyltransferase polysaccharide biosynthetic enzyme CspB, respectively. Both genes are predicted to directly play a role in polysaccharide biosynthesis.

A number of independent isolates of *L. delbrueckii* subsp. *bulgaricus*, obtained with either DCS or AMP selection, had mutations in a gene annotated as a putative Mg^2+^-transporting P-type ATPase. The finding that P-type ATPase derivatives were found with both types of antimicrobials indicates a secondary target mechanism. Despite an extra effort to understand the mode of action for the observed phenotypes, we can only speculate that reduced intracellular levels of manganese or eventually magnesium can alter the activity of enzymes crucial for cell envelope formation and indirectly lead to production of more capsular polysaccharide (CPS) or EPS. When nutrients are limited, formation of EPS and biofilm is induced and bacteria become highly tolerant to antibiotics (Mulcahy and Lewenza, 2011; Nguyen et al., 2011). Moreover, strains with higher extracellular polysaccharide production have been shown to have a significantly higher resistance to antibiotics (Arciola et al., 2005). Since most of the observed changes occur in genes encoding transport functions, cell wall formation, or stress responses, this potential link between stress, starvation, texture, and antibiotic resistance might be a hypothesis to explain the link between texture and antibiotic resistance in several of the derivatives found here.

Antibiotic resistance is considered to be one of the most important public health issues of our time, primarily due to the development of transmissible antibiotic resistance genes which can transfer between bacterial hosts. However, the use of cell envelope targeting antibiotics and antimicrobial agents to select the derivatives described here does not pose a threat to public health because the resistance is host specific and not based on transmissible antibiotic resistance genes.

The selective methods described in this study can therefore be used directly as an effective toolbox for strain improvement for the food industry.

## Data Availability Statement

The datasets presented in this study can be found in online repositories. The names of the repository/repositories and accession number(s) can be found in the article/supplementary material.

## Author Contributions

KS was involved in design of and performing the experiments, writing of the manuscript, and drawing of the figures. EJ was involved in design of the experiments and writing of the manuscript. IK was involved in writing of the manuscript and genome sequence analysis. All authors contributed to revising the manuscript.

## Conflict of Interest

All authors were employed by Chr Hansen A/S during the writing of this manuscript. Chr. Hansen A/S is a major commercial supplier of starter cultures to the fermentation industry. Additionally, some authors are shareholders in the company and/or inventors on patents and patent applications related to the described technology.

## Footnotes

1 http://www.ncbi.nlm.nih.gov/GenBank/

2 http://blast.ncbi.nlm.nih.gov/Blast.cgi
